# Assessing the Importance of Intraspecific Variability in Dung Beetle Functional Traits

**DOI:** 10.1371/journal.pone.0145598

**Published:** 2016-03-03

**Authors:** Hannah M. Griffiths, Julio Louzada, Richard D. Bardgett, Jos Barlow

**Affiliations:** 1 Lancaster Environment Centre, Lancaster University, Bailrigg, Lancaster, LA1 4YQ, United Kingdom; 2 Departamento de Biologia, Universidade Federal de Lavras, Lavras, Minas Gerais, 37200–000, Brazil; 3 School of Environmental Sciences, The University of Liverpool, Liverpool, 4 Brownlow Street, L69 3GP, United Kingdom; 4 Faculty of Life Sciences, Michael Smith Building, The University of Manchester, Oxford Road, Manchester, M13 9PT, United Kingdom; 5 Museu Paraense Emilio Goeldi, Av. Magalhães Barata, 376, Belém-Pará, Brazil; University of Oxford, UNITED KINGDOM

## Abstract

Functional diversity indices are used to facilitate a mechanistic understanding of many theoretical and applied questions in current ecological research. The use of mean trait values in functional indices assumes that traits are robust, in that greater variability exists between than within species. While the assertion of robust traits has been explored in plants, there exists little information on the source and extent of variability in the functional traits of higher trophic level organisms. Here we investigated variability in two functionally relevant dung beetle traits, measured from individuals collected from three primary forest sites containing distinct beetle communities: body mass and back leg length. In doing so we too addressed the following questions: (i) what is the contribution of intra vs. interspecific differences in trait values; (ii) what sample size is needed to provide representative species mean trait values; and (iii) what impact does omission of intraspecific trait information have on the calculation of functional diversity (FD) indices from naturally assembled communities? At the population level, interspecific differences explained the majority of variability in measured traits (between 94% and 96%). In accordance with this, the error associated with calculating FD without inclusion of intraspecific variability was low, less than 20% in all cases. This suggests that complete sampling to capture intraspecific variance in traits is not necessary even when investigating the FD of small and/or naturally formed communities. To gain an accurate estimation of species mean trait values we encourage the measurement of 30–60 individuals and, where possible, these should be taken from specimens collected from the site of study.

## Introduction

Understanding how biological diversity influences ecosystem processes is crucial if we are to predict and thus mitigate the consequences of anthropogenic driven species losses [1]. Functional diversity (FD) quantifies the value, range, and relative abundance of functional traits in a given ecosystem [2] and has been used to link biodiversity with a suite of ecosystem functions and services [3–9]. It has improved our understanding of species interactions and community assembly rules [10], as well as species responses to disturbance [11]. Additionally, it has been proposed that FD and its links to ecosystem processes could be of value for defining a planetary threshold for biodiversity loss [12,13]. Functional diversity, therefore, has the capacity to facilitate a mechanistic understanding of the impact anthropogenic disturbances on biological communities and the processes they govern [10], and could ultimately inform conservation management and policymaking decisions.

Functional traits (physiological, morphological or phenological characteristics measurable at the individual level that impact upon fitness; [14]) are the building blocks of FD indices and are generally calculated using mean trait values applied to all individuals of that species. This assumes that traits are ‘robust’, i.e. that greater variability exists between than within species [10,15–17]. There is, however, growing evidence that this is not always the case [10,18–22], especially when considering the traits of individuals originating from spatially discrete locations [21]. Furthermore, intraspecific trait variability is increasingly recognised as an important component of diversity driving ecosystem functioning [21] as well as functional responses to disturbances [23], and recent work has demonstrated that the failure to consider intraspecific trait variability in FD investigations has the potential to influence findings [24–26]. There is, therefore, a clear need to better understand the magnitude and source of variability in the traits of functionally relevant organisms [19,24,26].

It is often not feasible, or necessary, to gather information on every trait, from every individual within a given community [27]. Consequently, quantifying intraspecific trait variability [19,22,24] and understanding when and how it should be measured [15] has received reasonable attention in recent years. Concurrently, investigations have focussed on methods of incorporating within species variability into FD indices [28] and the impact of doing so for interpretation of results [26]. However, to our knowledge, this work has been exclusively carried out on plant traits [15,18,26,29], likely because the use of functional traits as a tool to investigate diversity-functioning relationships in non-producer systems is comparatively uncommon (but see [8,28,29]). Researchers adopting a trait-based approach using higher trophic level organisms must, therefore, make methodologically important decisions regarding the level of precision to employ without any empirical guidelines.

Here we investigated variability in invertebrate functional traits. Using data from a field-based biodiversity-ecosystem function experiment [8], where morphological measurements were collected from dung beetle individuals (n = 1962), we quantified the source and extent of variation in two functionally relevant traits: body mass and back leg length. In doing so, we ask the following questions: (i) what is the relative contribution of intra vs. interspecific variability in trait values; (ii) what sample size is needed to provide representative species mean trait values; and (iii) what impact does omission of intraspecific trait information have on the calculation of functional diversity indices from naturally assembled communities?

## Materials and Methods

### Field sites and sampling strategy

Sampling was carried out during July and August 2012 in the 17 000km^2^ landholding of Jari Florestal, located in the State of Pará in the north-eastern Brazilian Amazon (0°53S, 52°36W). Dung beetles were sampled from three *terra firme* primary forests (n = 30 beetle communities in each forest) as part of a biodiversity-ecosystem functioning experiment [8]; full permission was granted by the private land-owner, Jari Florestal, to carry out work at these sites, sampling did not involve any endangered species and permission to collect zoological material was granted to JL by the Instituto Brasileiro do Meio Ambiente e dos Recursos Naturais Renováveis (IBAMA). All sites were within 100km of one another, classified as dense lowland tropical rain forest, were subject to the same regional climatic conditions and contained distinct dung beetle communities (S1 Appendix for multidimensional scaling ordination plots and Multivariate Analysis of Variance of beetle communities and S2 Appendix for a map of the region and study sites).

Dung beetle communities were collected from within ninety 50 cm x 50 cm experimental plots (30 plots were arranged in a grid at each forest site, plots were separated by 100m within each grid) baited with a 100g mixture of 50:50 human and pig dung [30], protected from the rain by a plastic cover. After baiting the plots were left open for colonisation by beetles for either 12 or 24 hours. These opening times were selected to increase variation in the diversity in beetle communities that colonised the plots. Following colonisation, plots were closed to ensure beetles could not escape. Un-baited pitfall traps (13.5cm width, 9cm depth), buried flush with the ground surface and filled with salt and water were located inside each of the plots; these were opened when the plots were closed to capture the beetle communities following emergence from the soil. Experimental plots remained closed, and internal pitfall traps left in place for seven days in site 1 and site 3 but because logging operations in site 2 restricted access to the area, beetles were removed after fourteen days at this site. This difference in the time that beetles remained in the pitfall traps did not significantly reduce the body mass of beetles collected from site 2 (S3 Appendix for analysis of the effect of site on beetle biomass). When the plots were opened, beetles were collected from the pitfall traps and the soil beneath the plots was destructively sampled; beetles were also collected from this soil up to a depth of 50cm. More detailed sampling design and rationale are presented in [8].

### Trait selection and measurement

Beetles were identified to species level using a reference collection held at the *Universidade Federal de Lavras* (UFLA) in Brazil and region-specific classification keys developed by F.Z. Vaz-de-Mello and T.A. Gardner (unpublished). Using traits to inform biodiversity-ecosystem functioning investigations involves defining the function of interest, identifying predictive traits for that function, and gathering representative values for those traits [31]. The ecological functions provided by dung beetles result from the burial of mammalian dung [32]. We therefore measured morphological traits relevant to excavation and burial [33] from every individual (n = 1962); namely pronotum volume (pronotum area multiplied by pronotum height), front leg area, the ratio of back to front leg lengths (S4 Appenidix for example of these morphological measurements; measured using a Leica M250 microscope and Life Measurement software); and dry body mass (determined using a Shimatzu AY220 balance with precision to 0.0001g). Body mass, the ratio of back to front leg lengths, body mass adjusted pronotum volume and body mass adjusted front leg area were used previously to create multi-trait FD indices and successfully predict seed burial and dispersion throughout the soil profile [8]. We therefore selected these traits for use in this study. However, because the non-body mass-adjusted traits are co-linear (S1 Fig) we present results from the two least correlated traits in the main text: body mass and back leg length (Pearson’s ρ = 0.89). Analyses on all other results are detailed in S5 Appendix.

Sixty-one species and morphospecies were recovered during sampling; the abundance of each varied from 1–239 individuals. However, in order to assess the magnitude and source of variability of measured traits, we selected only the species from the complete dataset for which we collected 50 or more individuals (n = 13).

### Statistical analyses

All analyses were carried out in R version 3.0.2 [34]. The first aim of this investigation was to quantify the extent and source of variability (intra vs. interspecific) in dung beetle functional traits. To do this, we performed variance component analyses following methods presented by Messier, McGill & Lechowicz (2010) [18]. Each trait was log_10_ transformed to normalise the data and general linear mixed models (lme) from the ‘nlme’ package [35] were fitted to the variance within and between species. These models contained no fixed effects; individual was nested within species and these were included as random factors. A variance component analysis (varcomp) from the ‘varComp’ package [36] was performed on each model.

Our second objective was to determine the number of individuals from which measurements should be taken in order to provide a representative value for each dung beetle functional trait. This was achieved through resampling (with replacement) all individuals of the thirteen species for which we had a sample size of n ≥ 50, to create sub-sets containing 3 to 100 individuals for each species (n = 1000 per sub-set). This was possible up to a sub-set size of 50 individuals for every species, but where the target sub-set size was larger than the number of individuals collected for a particular species, re-sampling was stopped. From each resampled dataset the standard error (SE) of each trait was calculated and from these we created a mean SE for each sub-set size. These mean standard error values were compared to the overall mean trait value calculated using every individual in the dataset for each trait and each species. The number of individuals needed to create a mean standard error within 5% of the overall sample mean was considered the minimum necessary to provide a representative trait value. This threshold value was chosen based on the 95% confidence limits commonly used in frequentist statistics. To assess if sample size can be reduced when considering a single population, this process was repeated but using only individuals collected from one of the experimental sites. As with the analysis using all individuals from each species, resampled sub-sets of individuals from just one site contained a minimum of 3 individuals and a maximum of 100. Site was selected for each species based on where they occurred in the greatest abundance (S6 Appendix for species abundances at each site). The mean standard errors generated during resampling were compared to site-specific species mean trait values.

The final goal of this study was to better understand how omission of intraspecific trait variability influences functional diversity indices when assessing naturally formed communities. Our focal traits were used previously to calculate multi-dimensional functional diversity indices [8]. However, in a plant-based investigation, Albert *et al*. (2010) [25] demonstrated that functional traits are likely to display unequal variance. Combining multiple traits together to calculate multidimensional indices could, therefore, mask the differences in traits and species that we are seeking to better understand [24]. Consequently, for the purposes of this study we calculated functional diversity using two single trait indices: community weighted mean (CWM) and functional richness (FRic). Community weighted mean is the mean value of a trait within a community, weighted by the relative abundances of the species carrying that trait [14,37]. FRic describes the volume of functional trait space occupied by a community; when using single traits it is the range in values [38].

These two indices were calculated twice for each community, once using individual trait values from each beetle captured within experimental plots (inclusion of intraspecific trait variability) and subsequently using mean species trait values (omission of intraspecific trait variability). We carried out these analyses using mean trait values because they are most commonly used in the calculation of FD indices (e.g. [24]). Following methods presented in Lavorel *et al*. (2007) [39], when calculating CWM traits with the inclusion of intraspecific variability, we calculated a mean for each community using values measured from each individual. Linear regressions were performed to assess the relationships between FD indices calculated with and without the inclusion of intraspecific trait information. R^2^ values from these models provide the percentage of information excluded from the FD indices when intraspecific trait information is omitted [26].

## Results

### Extent of trait variability

**Fig 1:**
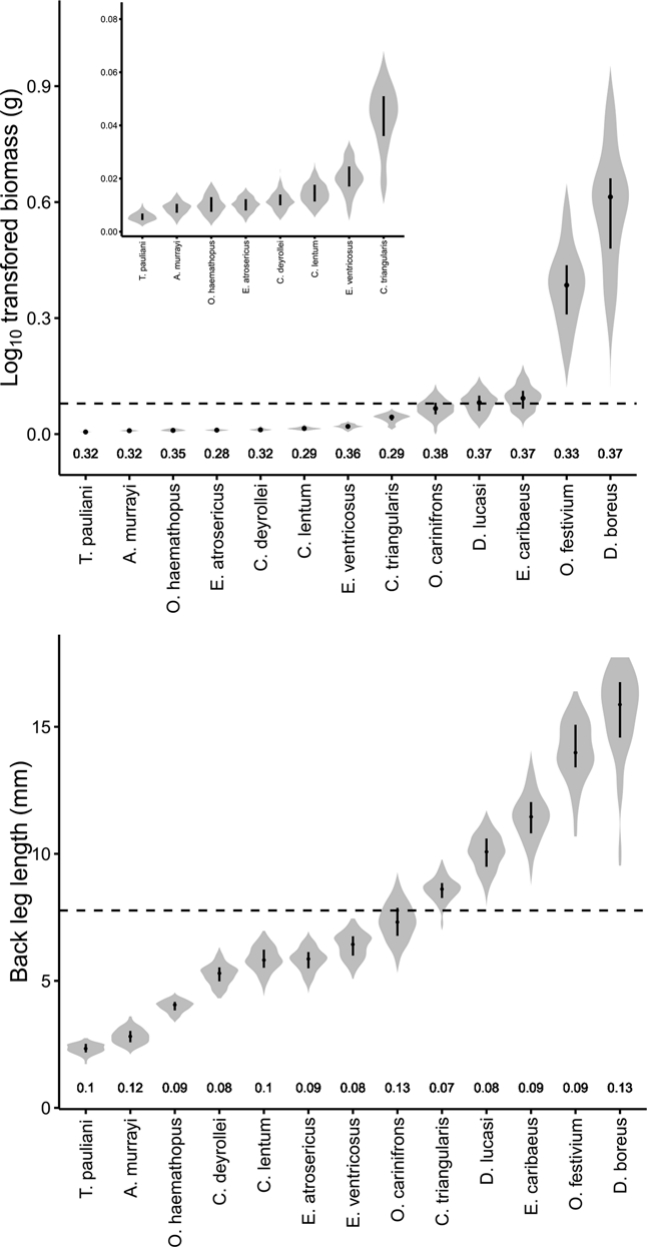
**Extent of intraspecific variability in dung beetle body mass (a) and back leg length (b).** Violin plots display (i) the density of data estimated by kernel method (grey areas); (ii) the median value (black horizontal dots in the centre of violins); and (iii) the interquartile range (between the top and bottom of the vertical black lines). Results are presented by species, ordered by their mean trait values and the coefficients of variation are given for each species below the violin. Horizontal dashed lines on each panel show the mean body mass and back leg length value (0.079g and 7.77mm respectively) of all species collected during sampling (61 species).

The complete measurement of body mass, pronotum volume, front leg area and back and front leg lengths from all 1962 dung beetle individuals amounted to around 240 hours of researcher time. We found large interspecific variability across both the body mass and back leg lengths of the thirteen focal species studied (Fig 1). Species mean values ranged from 0.005g to 0.804g for body mass (FRic = 0.779g; Fig 1(A)) and from 2.32mm to 15.59mm for back leg length (FRic = 13.27mm; Fig 1(B)). When individual, rather than mean trait values were considered, variability increased by 87.03% for body mass, ranging from 0.003g to 1.460g (FRic = 1.457g) and by 21.70% for back leg length, ranging from 1.68mm to 17.83mm (FRic = 16.15mm). This greater influence of intraspecific variability on the range in body mass values is reflected in differences in the coefficients of variation (CV: standard deviation divided by the mean) for both traits. The mean CV of all species for body mass was consistently larger than that of back leg length; 0.33 compared to 0.1, respectively (Fig 1).

### Source of trait variability

The partitioning of variance in the two traits revealed interspecific variance accounted for the vast majority of variability compared to intraspecific differences. Interspecific differences were responsible for 94% and 96% of variability for body mass and back leg length respectively, whereas intraspecific variation accounted for just 5% and 3% for body mass and back leg length.

### Sample size selection

**Fig 2:**
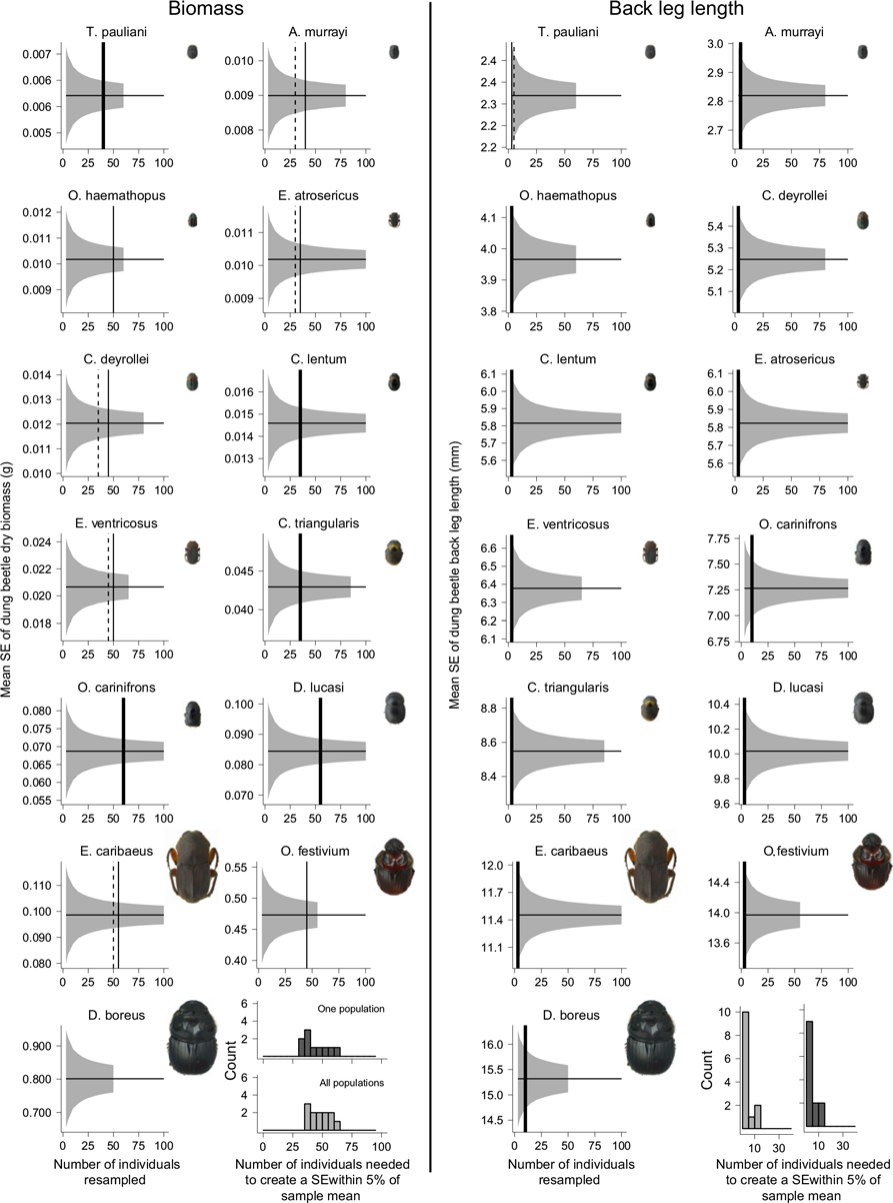
Resampling of dung beetle body mass and back leg length. Total population mean (solid horizontal black lines; calculated using all individuals from each species, n = 51–229) and mean standard error (grey ribbons; calculated using resampled data from focal species, n = 13, collected from all sampling locations) of dung beetle dry body mass (left panel) and back leg length (right panel) with species photographs. Photographs are scaled to each other; smallest species, *Trichillum pauliani*, length: 5.5mm; largest species, *Dichotomius boreus*, length: 24mm length. Species trait values were resampled to create new datasets containing 3 to 100 individuals and the mean standard error was calculated from the new datasets. Vertical lines indicate the number of individuals needed to create a mean standard error within 5% of the total population mean when considering individuals from every site (thin solid lines), one site only (dashed lines). When there was no difference in the numbers needed between all sites and one site, thick solid lines are used. The body mass panel for *D*. *boreus* has no vertical lines because resampling was stopped at a sub-set size of 50 individuals (the sample size of this species), which was before the mean SE had fallen within 5% of the total sample mean. Histograms display the frequency with which each sample size created a mean standard error below the 5% threshold using individuals from all site (light grey) and one site (dark grey). Results are presented by species, ordered by their mean trait values.

Between 35 and 60 individuals were needed to reduce the mean standard error (SE) of body mass to within 5% of the total sample mean when individuals from all three sampling locations were included in resampling (Fig 2). When analyses were repeated using individuals from just one sampling site, 5 or 10 fewer individuals were required for 5 of the focal species (dashed lines Fig 2). This resulted in between 30 and 60 individuals needed to attain an accurate estimate of the population mean. When considering beetles from one sampling site, or all three, 35 individuals was the most frequently required sample size (Fig 2). The mean SE of back leg length fell to within 5% of the total sample mean when considering just 3 individuals for the majority of species (10 out of 13; Fig 2). Examining just one population did not reduce the number of individuals required to accurately estimate mean leg length in any species.

### The influence of intraspecific trait variability on functional diversity indices

**Fig 3:**
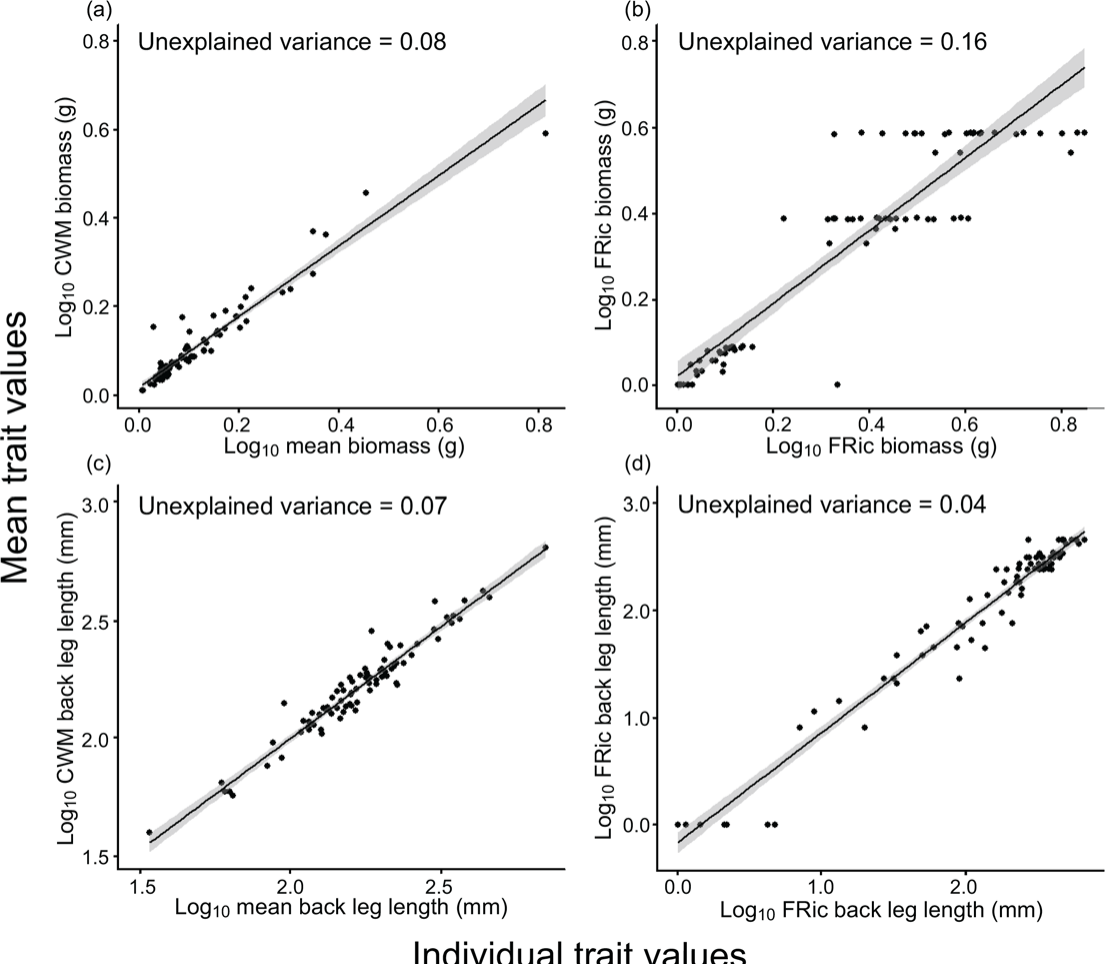
Associations between functional diversity indices calculated with (x–axis) and without (y–axis) the inclusion of intraspecific trait variability. Community weighted mean (CWM) of body mass (a), functional richness (FRic) of body mass (b), CWM back of back leg length (c) and FRic of back leg length (d). Linear model outputs are displayed: regression lines (solid back lines), standard errors (grey ribbons) and the inverse of R^2^ values to describe the loss of information as a result of exclusion of intraspecific trait information.

The error associated with calculating CWMs without considering intraspecific trait information was 8% and 7% for body mass and back leg length respectively (Fig 3(A) and 3(C)). Calculating FRic without including individual trait variability resulted in 16% and 4% loss of information for body mass and back leg length (Fig 3(B) and 3(D)). The strength of relationships between the indices calculated with and without intraspecific trait variability were consistently weakest when considering body mass.

## Discussion

Our study has taken the first steps in quantifying the importance of variability in invertebrate traits for the calculation of functional diversity (FD) indices. In doing so, we reveal that the dung beetle traits we examined displayed much greater inter- than intraspecific variability at the spatial scale of this investigation. This resulted in small errors when using mean trait values to calculate single trait functional diversity indices compared to using individual trait values. Our results therefore support the use of mean trait values to summarise species trait information when considering trait diversity of invertebrate communities sampled at relatively small geographic scales.

### Are dung beetle functional traits robust?

We tested the assumption that dung beetle functional traits are robust, i.e. that they vary more between than within species [17]. Intraspecific differences in trait values were responsible for between 2.6% and 5% of total variability for pronotum volume (S5 Appendix; Fig 3) and body mass, respectively. We are therefore confident that the assumption of robust traits in dung beetles is valid, at least within geographically close primary forest sites (i.e. 90 km) located within the same interfluvium. However, we recognise that the contribution of intraspecific differences in trait values could increase with increasing spatial scales or along large environmental gradients. Therefore, to more thoroughly test our assertion, further work is needed to quantify variability in traits derived from individuals originating from geographically distant sites, as well as sites distributed along longer gradients of environmental conditions, including anthropogenic disturbance [11].

Our conclusion that dung beetle functional traits vary more between than within species is in contrast to a number of plant based studies that report greater [21], equal [18] or less (but non-negligible) [19,25] contributions of intra, compared with interspecific variability [22]. Although these studies were conducted over larger spatial scales, with differences in sampling strategies, there are well-established biological reasons as to why the traits of animals should display less intraspecific variability than those of plants. Namely, most animals can move in response to environmental cues or pressures whereas plants cannot. Therefore, many plant species can quickly respond physiologically to changes in, for example, resource availability [40,41]. Phenotypic plasticity (the capacity of a given genotype to adopt different phenotypes under varying environmental conditions; [42]) in morphological/physiological traits is therefore likely to be of a greater evolutionary advantage in sessile plants than in mobile animals.

Although not specifically tested, our findings caution against the categorisation of continuous traits in dung beetles and other invertebrates, unless the distribution of values within a community show clearly discrete clusters of species within which a threshold can be reasonably placed. This is because the mean trait value of one species can frequently represent a small or large value of an individual from a species of a similar size, which is apparent from consideration of the violin plots. Furthermore, *O*. *carinifrons*, *D*. *lucasi and E*. *caribaeus* all display body mass values that traverse the mean value of all species collected, while individuals of *O*. *carinifrons* and *C*. *triangularis* have back leg length values that span the population mean. A number of previous dung beetle functional diversity investigations have categorised species as small or large based on thresholds such as body length [43–45] or ability to fit through a certain size mesh [46,47]. Our threshold value (the mean body mass all species) artificially categorises three species as either large or small when in fact individuals have a high probability of displaying trait values that places them in a different category. Therefore, gathering species into groups artificially imposes a discrete structure on functional differences that are generally continuous, resulting in loss of information [48]. This could ultimately compromise efforts to determine patterns between organisms and the ecosystem processes they govern if the miss-categorisation of individuals involved leads to an underestimation of relationships.

### How many traits are enough?

We have demonstrated that intraspecific trait variability in dung beetle traits is negligible, suggesting that average values should accurately represent species functional characteristics. But how many individuals per species should be assessed to provide a realistic estimation of the actual sample mean, whilst minimising sampling effort? Our results suggest that this depends on the trait and species of interest. Body mass was the most variable, and consequently the trait from which most individuals must be measured in order to provide a reliable mean. For the majority of species, we found that it was necessary to measure between 35 and 55 individuals to reduce the standard error of biomass to within 5% of the total sample mean, whereas between just 3 and 10 individuals were required for back leg length. Considering individuals from one sampling location reduced the sample size required for 5 species by 5 or 10 individuals for body mass, but had no impact on the numbers needed for back leg length. This implies that caution is needed when designing a sampling regime based on the assumption that individuals will display less intraspecific trait variability if originating from the same population. While this may be true (for some traits), it appears that this does not necessary translate into a large reduction in the numbers of individuals needed to gain a representative estimate of mean trait values. These results suggest that investigators should measure at least 30 individuals when working with invertebrate traits that are likely to display high levels of phenotypic plasticity.

An explanation for the different levels of variability in traits and the sample size required to reduce their standard error to within 5% of the total sample mean could lie in differing levels of plasticity displayed by each trait. Body mass can change in response to short term environmental cues, and as such it displays phenotypic plasticity [42]. Morphological characteristics such as leg length or leg area are, however, determined during larval development [49] and are fixed during adult life. Thus, fluctuations in resources over very small spatiotemporal scales (e.g. weeks or kilometres) would have little impact on the variability of these fixed traits compared with body mass. This is supported when considering pronotum volume and front leg area (S5 Appendix), both of which, like back leg length, are non-plastic traits in adult beetles. The number of individuals needed for these traits to reduce variability to within 5% of the total sample mean was also fewer than was needed for body mass.

These findings suggest that the sampling of invertebrate traits from all individuals collected during an ecological investigation is not necessary. However, if dealing with small populations, complete sampling may not represent significant increases in time investments, but will increase the accuracy with which the organisms are described. Furthermore, detailed data such as these will be useful for the creation of large-scale trait databases and would allow future investigation into the relative importance of intraspecific variability of individuals originating from geographically distant sites. Trait databases (e.g. [50]) are increasingly important tools in facilitating large-scale functional investigations in plant-focussed studies (e.g. [51]) but equivalent trait collections are lacking for higher trophic level organisms. Further work is therefore needed to understand the ability of plastic versus fixed traits to predict animal-mediated ecosystem functioning. This would facilitate the targeted development of much-needed trait databases for non-producer organisms.

### The influence of intraspecific trait variability on functional diversity indices

Our final objective was to assess how the omission of intraspecific information in dung beetle traits influenced the accuracy with which functional diversity indices described naturally formed communities. Intraspecific variability contributed very little (less than 5%) to overall community level trait variability. Therefore, perhaps unsurprisingly, omission of within species differences in trait values only led to the loss of small amounts of information when calculating functional diversity indices; less than 20% for all traits and indices. For all traits CWM was more sensitive to the omission of intraspecific trait variability than FRic. This is incongruent with the findings of Albert *et al*. (2012) [26] who report CWM to be less sensitive than FRic to the exclusion of intraspecific variability. Albert *et al*. (2012) [26] calculated FD of single traits with and without varying levels intraspecific differences for communities consisting of between 22 and 51 species, covering an area of 1% - 87% of the sampling plots. In contrast, this investigation considered communities containing between 1 and 11 species with abundances of between 1 and 95 individuals. It is feasible, therefore, that the differences we see in the sensitivity of the indices to the exclusion of intraspecific trait information between this study and that of Albert *et al*. (2012) [26] could arise from a reduction in precision of metrics as abundance and/or species decreases; a possibility that has not been previously explored. Further work on which index performs best under contrasting community sizes and levels of diversity will provide useful guidelines for the investigator faced with the choice of multiple functional diversity indices (e.g. [52]).

## Conclusion

Our exploration into the sources of trait variability in a functionally important invertebrate group has demonstrated that using dung beetle mean trait values when dealing with individuals from the same geographic region is likely the most ecologically meaningful approach [15]. To accurately estimate mean trait values, however, we urge thoughtful consideration of the variability of the focal traits and the sampling location(s) from which individuals are collected. We reveal that even when considering small communities of low species richness and/or abundances, failure to incorporate intraspecific trait variability does not result in the loss of large amounts of information. However, our results show that to ensure accurate estimation of invertebrate mean trait values for use in functional diversity indices, the measurement of at least 30 individuals is necessary. Increasing the precision with which the functional traits of organisms are described within an environment will increase the accuracy with which biological diversity can be linked to ecological processes. The importance of functional diversity is increasingly recognised as a tool for predicting the consequences of human impacts on ecosystems [13,53], and functional traits are the fundamental building blocks of this fast developing field. Developing a better understanding of the ecological importance of intraspecific variance in trait values will help develop functional ecology into a more precise, quantitative and predictive science [10].

## Supporting Information

S1 Appendix Multidimensional scaling ordination plots of dung beetle communities and multivariate analysis of variance. (DOCX)

S2 Appendix Map of study sites. (DOCX)

S3 Appendix Analysis of site on dung beetle body mass values. (DOCX)

S4 Appendix Dung beetle morphological trait measurements. (DOCX)

S5 Appendix Analyses carried out on pronotum volume and back leg length. (DOCX)

S6 Appendix Species abundances at each site. (DOCX)

S1 Data Excel file of data used in this paper. (XLSX)

S1 Fig Associations between raw traits. (DOCX)
