# Supplementary material for: Assessing the Importance of Intraspecific Variability in Dung Beetle Functional Traits
Source: PLoS One. 2016 Mar 3;11(3):e0145598. doi: 10.1371/journal.pone.0145598 (PMC4777568; doi:10.1371/journal.pone.0145598)
Supplement: S1 Appendix — (DOCX) [file pone.0145598.s001.docx]

We performed a MANOVA (multivariate analysis of variance) on nonmetric multidimensional scaling (NMDS) ordinations of the dung beetle communities originating from site 1 (open circles); site 2 (closed circles); and site 3 (crosses). This revealed that site significantly affected the community structure of the dung beetles used in the investigation (F _2, 57_ = 12.2, *P* = 0.01).
