# Supplementary material for: Assessing the Importance of Intraspecific Variability in Dung Beetle Functional Traits
Source: PLoS One. 2016 Mar 3;11(3):e0145598. doi: 10.1371/journal.pone.0145598 (PMC4777568; doi:10.1371/journal.pone.0145598)
Supplement: S3 Appendix — (DOCX) [file pone.0145598.s003.docx]

*E. caribaeus* and *D. lucasi* were the only species from which we collected sufficient individuals from each forest site (n = 15) to analyse how body mass was the effected by sampling location, and thus time in that beetles remained in pitfalls. Where site significantly affected body mass values, Tukey’s HSD tests were performed to assess the source of the differences. *E. caribaeus* and *D. lucasi* differed significantly across sites (F_2, 137_ = 17.47, *P* < 0.0001 and F_2, 226_ = 10.76, *P* < 0.0001 respectively, Fig. S3.1). Post-hoc tests revealed that the body mass of *E. caribaeus* differed significantly between site 1 and site 3 (*P* < 0.0001) and between site 2 and site 3 (*P* < 0.0001); body mass was significantly higher at site 3 than at site 1 and site 2. The body mass of *D. lucasi* was significantly higher at site 3 than site 2 (*P* < 0.0001). If the extra time that beetles remained in pitfalls at site 2 compared to site 1 and 3 had led to increased decay, it would be expected that the body mass of beetles at this site would be consistently lower.

**Figure S3.1. Sampling site differences in dung beetle body mass.** Medians (central vertical lines) interquartile ranges (boxes) and outliers (black points) are displayed for species collected from site 1 (white boxes); site 2 (light grey boxes) and site 3 (dark grey boxes).
