# Supplementary material for: Assessing the Importance of Intraspecific Variability in Dung Beetle Functional Traits
Source: PLoS One. 2016 Mar 3;11(3):e0145598. doi: 10.1371/journal.pone.0145598 (PMC4777568; doi:10.1371/journal.pone.0145598)
Supplement: S4 Appendix — (DOCX) [file pone.0145598.s004.docx]

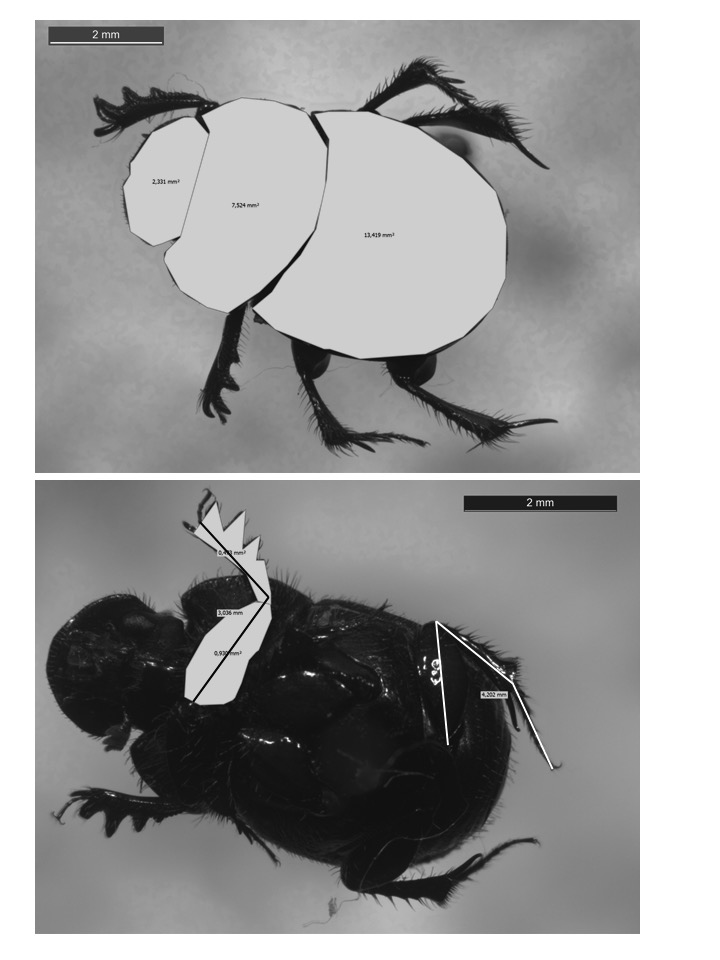


Dung beetle trait measurements collected using Leica M250 microscope and Life Measurement software. Dorsal measurements (top panel): head area, pronotum area, elytra area; ventral measurements (bottom panel): front and back leg length, femur area, tibia area. The scale bar represents 2mm, the species shown is *Cathidium deyrollei.*
