# Supplementary material for: Assessing the Importance of Intraspecific Variability in Dung Beetle Functional Traits
Source: PLoS One. 2016 Mar 3;11(3):e0145598. doi: 10.1371/journal.pone.0145598 (PMC4777568; doi:10.1371/journal.pone.0145598)
Supplement: S5 Appendix — (DOCX) [file pone.0145598.s005.docx]

**Figure**. **S6**.**1**. Extent of intraspecific variability in dung beetle pronotum volume and front leg are for thirteen dung beetle species collected from the north-eastern Brazilian Amazon, state of Pará. Violin plots display (i) the density of data estimated by kernel method (grey areas); (ii) the median value (black horizontal lines in the centre of violins); and (iii) the interquartile range (between the top and bottom of the vertical black lines). Results are presented by species, ordered by their median trait values and the coefficients of variation are given for each species below the violin.

VARIANCE PARTITIONING

Interspecific differences were responsible for 96.6% and 96.7% of variability for pronotum volume and front leg area respectively, whereas intraspecific variation accounted for just 2.6% in both traits.

**Figure. S6.2.** Total population mean (solid horizontal black lines; calculated using all individuals from each species (n = 51 - 229) and mean standard error (grey ribbons; calculated using resampled data) of dung beetle pronotum volume and front leg area with species photographs. Photographs are scaled to each other; smallest species, *Trichillum pauliani,* length: 5.5mm; largest species, *Dichotomius boreus,* length: 24mm length. Data were resampled (with replacement) to create new datasets containing 3 to 100 individuals (n = 1000 datasets per sample size) and the mean standard error was calculated from the new datasets. Vertical dashed back lines on each panel show the number of individuals needed to create a mean standard error within 5% of the total population mean. Histograms in the bottom right corners display the frequency with which each sample size created a mean standard error below the 5% threshold. Results are presented by species, ordered by their median trait values.

**Figure. S6.3.** Associations between single trait functional diversity indices calculated without the inclusion of intraspecific trait variability: community weighted mean (CWM) pronotum volume (a), functional richness (FRic) of pronotum volume (b), CWM front leg area (c) and FRic front leg area (d). Linear model outputs are displayed: regression lines (solid back lines), standard errors (grey ribbons) and the inverse of R^2^ values to describe the loss of information as a result of exclusion of ITV information.
