# Supplementary figures and images for: Assessing the Importance of Intraspecific Variability in Dung Beetle Functional Traits
Source: PLoS One. 2016 Mar 3;11(3):e0145598. doi: 10.1371/journal.pone.0145598 (PMC4777568; doi:10.1371/journal.pone.0145598)

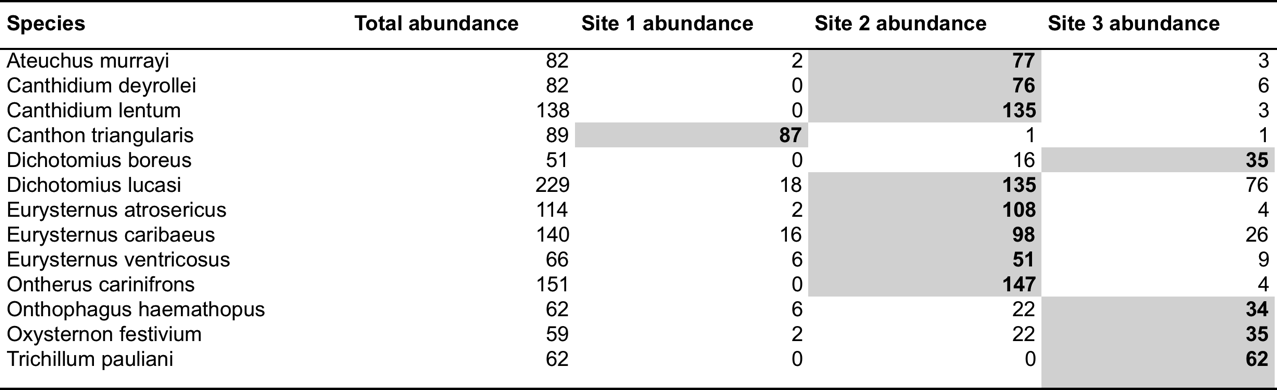

Supplement: S6 Appendix — (DOCX) [file pone.0145598.s006.docx]
