# Supplementary material for: Assessing the Importance of Intraspecific Variability in Dung Beetle Functional Traits
Source: PLoS One. 2016 Mar 3;11(3):e0145598. doi: 10.1371/journal.pone.0145598 (PMC4777568; doi:10.1371/journal.pone.0145598)
Supplement: S1 Fig — (DOCX) [file pone.0145598.s008.docx]

**Associations between dung beetle functional trait values**: bivariate plots (lower panels), distributions (diagonal), and Pearson’s ρ (upper panels). Biomass and pronotum volume were cube-rooted, and front leg area square-rooted to convert values to the same scale.
